# Systematic Review of Survival Analysis in Leprosy Studies—Including the Following Outcomes: Relapse, Impairment of Nerve Function, Reactions and Physical Disability

**DOI:** 10.3390/ijerph191912155

**Published:** 2022-09-26

**Authors:** Celivane Cavalcanti Barbosa, Gilberto Silva Nunes Bezerra, Amanda Tavares Xavier, Maria de Fátima Pessoa Militão de Albuquerque, Cristine Vieira do Bonfim, Zulma Maria de Medeiros, Wayner Vieira de Souza

**Affiliations:** 1 Department of Collective Health, Aggeu Magalhães Institute, Oswaldo Cruz Foundation, Recife 50740-465, Brazil; 2 PRISM Research Institute, Technological University of the Shannon, Midlands Midwest, N37 HD68 Athlone, Ireland; 3 Postgraduate Health Sciences, University of Pernambuco, Recife 50100-130, Brazil; 4 Department of Parasitology, Aggeu Magalhães Institute, Oswaldo Cruz Foundation, Recife 50740-465, Brazil; 5 Postgraduate Program in Public Health, Federal University of Pernambuco, Recife 50670-901, Brazil; 6 Social Research Division, Joaquim Nabuco Foundation, Ministry of Education, Recife 52171-010, Brazil

**Keywords:** leprosy, epidemiology, statistical analysis, survival analysis

## Abstract

Leprosy is a public health problem in South American, African and Oceanian countries. National programs need to be evaluated, and the survival analysis model can aid in the construction of new indicators. The aim of this study was to assess the period of time until the outcomes of interest for patients with or exposed to leprosy by means of survival analysis surveys. This review researched articles using the databases of PubMed, Science Direct, Scopus, Scielo and BVS published in English and Portuguese. Twenty-eight articles from Brazil, India, Bangladesh, the Philippines and Indonesia were included. The Kaplan–Meier method, which derives the log-rank test, and Cox’s proportional hazards regression, which obtains the hazard ratio, were applied. The mean follow-up until the following outcomes were: (I) leprosy (2.3 years) in the population who were exposed to it, (II) relapse (5.9 years), (III) clinical manifestations before, during and after treatment—nerve function impairment (5.2 years), leprosy reactions (4.9 years) and physical disability (8.3 years) in the population of patients with leprosy. Therefore, the use of survival analysis will enable the evaluation of national leprosy programs and assist in the decision-making process to face public health problems.

## 1. Introduction

Leprosy is considered a neglected and infectious disease [1,2]. The etiologic agent is *Mycobacterium leprae*, an obligate intracellular bacterium with an affinity for the peripheral nervous system [3]. In general, people affected by the disease suffer social and psychological repercussions due to deformities and physical disabilities caused by the disease’s progression [4,5].

The global prevalence rate of leprosy has reached less than one case per 10,000 inhabitants, but it is considered a public health problem in countries located in South America, Africa and Oceania, which have not yet achieved the elimination of the disease [6]. In 2020, 127,558 new cases were detected worldwide, of which 74.0% were located in Brazil, India and Indonesia [6], and grade 2 of physical disability was diagnosed in 7198 (5.6%) individuals in 64 countries [6]. It is understood that there is a need to carry out studies to estimate the time between the date leprosy was diagnosed and the date of discharge, as well as to analyze the risk factors related to the outcome in search of its elimination.

The survival analysis model can help in interpretations about the evolution of diagnoses and treatments, collaborating with the description of the behavior of leprosy and the prognostic factors related to it [7,8]. Survival analysis begins by counting the mean follow-up from the initial observation until the outcome of interest [9,10]. The epidemiological studies that can be used are experimental (clinical trials) and observational cohorts [11,12].

Given that leprosy continues to be a secular public health problem, and further development of the analysis is still a challenge, carrying out a systematic review was chosen as a form of contribution. This review sought to pursue the scientific production of survival analysis studies in patients with leprosy or those who have exposed to leprosy. Thus, the objective was to assess the period of time until the outcomes of interest, which may constitute indicators to evaluate the results of the disease control program in the future.

## 2. Materials and Methods

### 2.1. Registration Protocol

In this systematic review, we used the guidelines and checklist (Table S1) from the PRISMA 2009 preferred reporting items for system reviews and meta-analyzes [13] and the predefined protocol prospective international registry platform for systematic reviews [CRD42022296026] [14].

### 2.2. Data Sources and Research Strategy

The bibliographic survey of electronic databases was carried out in February 2022 without restrictions on the publication date and location. The databases used were PubMed (National Center for Biotechnology Information), Science Direct (Elsevier), Scopus (Elsevier), Scientific Electronic Library Online (SciELO) and the Virtual Health Library (BVS) for journal articles published in English and Portuguese, aiming to cover studies carried out in locations where the incidence of leprosy is higher. The keywords used for the searches were “Leprosy”, “Mycobacterium leprae”, “Survival”, “Survival Analysis”, “Survival Rate, Proportional Hazards Models” and “Kaplan–Meier Estimate”. These keywords were researched in various forms of combinations. The search strategy performed in PubMed was as follows: (((“Leprosy” [Mesh]) AND (Lepros *)) OR (“Mycobacterium leprae” [Mesh])) OR (“Mycobacterium leprae”)) AND (“Survival Analysis” [Mesh]). In addition, the references of eligible articles were consulted through manual searches (hand-searching).

### 2.3. Study Selection and Data Extraction

The articles were selected by two independent reviewers (CCB and ATX) initially by reading the titles and abstracts; some were ineligible for not meeting the inclusion criteria. After the removal of studies that did not meet the criteria and removing duplicates, the full texts were read in order to carry out a new eligibility certification. Based on the inclusion criteria of the study, data were extracted using a data extraction form and, in the case of any lack of consensus, an experienced third reviewer (CVB) would make her assessment. The inclusion criteria were: (I) survival studies on patients with or exposed to leprosy; (II) the mean follow-up until the occurrence of any type of outcome of interest, for example: cure, relapse, reactive episodes, degree of physical disability, illness in household and non-household contacts; (III) application of survival curves using the Kaplan–Meier method (estimates the conditional probability between time and outcome) and/or the regression model (estimates the effect of predictor variables using the risk function). The outcomes were grouped and counted in order to address the five with the highest frequencies in the discussion. Articles were eliminated if they did not meet the inclusion criteria, were not in English or Portuguese, literature reviews, systematic articles with or without meta-analysis or book chapters, reviews, conference abstracts and letters to the editor.

The data were analyzed qualitatively and displayed in the form of tables and narrative text. The following items were extracted from each selected study: name(s) of author(s), year of publication, country, study design, duration of follow-up (years), study population, outcome of interest, number of patients/population and number of events, mean follow-up, incidence density, survival curve methods, comparison of survival curves, regression and measure of association. For the five main outcomes listed by high frequency, the following data were extracted for both paucibacillary and multibacillary: name(s) of author(s), year of publication, mean follow-up (years), incidence density/100 person-years and its 95% confidence interval (CI), Kaplan–Meier curve interpretations in years, hazard ratio and its 95% CI. This extraction was performed independently by two reviewers (CCB and ATX), and in the presence of any discrepancy, a third reviewer (CVB) verified the data.

### 2.4. Risk of Bias and Quality Assessment

The assessment of risks of bias and study quality was performed by two independent reviewers (CCB and ATX) using the Standard Quality Assessment Criteria for Evaluation of Primary Research Papers from a Variety of Fields [15]. The tool consists of 14 items to assess the methodological quality of each study applied in a systematic review. Each item received a score according to the response, such as “0” for negative responses, “1” for partial responses and “2” for positive responses. In this manner, the scores were performed as follows: (I) maximum points are based on 28—(2 × number of N/A), (II) total points are calculated by adding the total amount of points obtained from all items, and (III) the summary score is represented by the total score obtained divided by the maximum score presented in percentage. In the summary score, articles with a percentage above 75% were considered better quality, and articles with a percentage below 55% were considered low quality. Therefore, the higher the final percentage, the lower the risk of bias and the better the quality of the study. Any lack of consensus was discussed with a third reviewer (CVB).

## 3. Results

### 3.1. Flow Diagram of Included Studies

**Figure 1:**
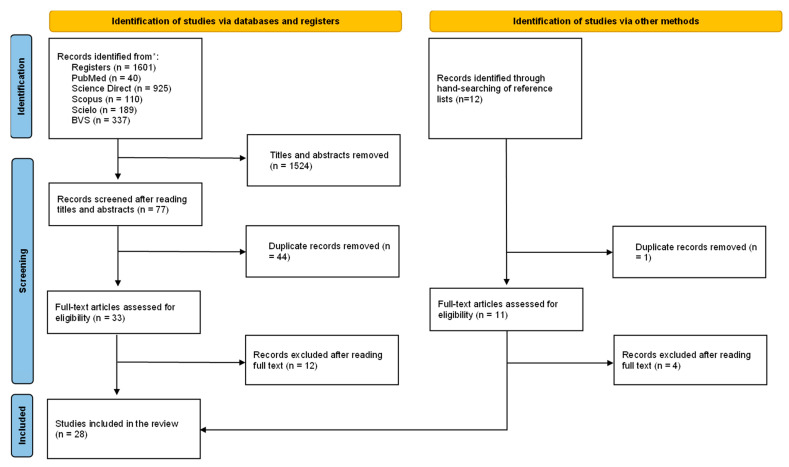
Flow diagram systematic search and review process. * Reporting the number of records identified from each database.

The search generated 1601 results (PubMed—40, Science Direct—925, Scopus—10, Scielo—189, VHL—337), of which 1524 were removed after screening titles and abstracts. Of the 77 that remained, 44 duplicates were removed. Subsequently, 33 articles were evaluated based on their full text, with 12 being removed for not meeting the inclusion criteria. Finally, 21 studies were included in this review (Figure 1). 

In the manual search, 12 articles were found, one duplicate was removed and after reading the full text, four articles were removed because they did not meet the inclusion criteria. As a result of the manual search, seven articles were included. The total number of articles included was 28, as detailed in the PRISMA (preferred reporting items for system reviews and meta-analyses) flow diagram shown in Figure 1.

### 3.2. Study Description

All 28 studies included in this systematic review are described in Table 1.

The studies were published between 1994 and 2022, which is equivalent to 28 years of scientific research addressing the topic of survival analysis and leprosy. The years that stood out were 2000, 2008 and 2012, with three articles each, and especially 2021, with four. Among the study designs, the cohort type predominated (19; 67.9%), being six (21.4%) prospective, five (17.9%) retrospective and eight (28.6%) unclassified. The follow-up duration of the studies averaged 9.5 years. Regarding the location of the studies, the highlighted countries were Brazil (11; 39.3%), India (6; 21.4%), Bangladesh (5; 17.9%), the Philippines (2; 7.1%) and Indonesia (2; 7.1%) (Table 1).

Concerning the study population, the studies were divided into leprosy contacts (six; 21.4%). Of these, three studies were selected with intra-household and extra-household contacts. The other study population was leprosy patients (22; 78.6%), and in 10 studies, multibacillary leprosy patients were selected (Table 1). 

Within the population of exposed patients and those with leprosy, 12 events of interest were found, and a single study may have up to four outcomes. In the population of leprosy contacts, the outcome was leprosy, and in the population of leprosy patients, the main events of interest were relapse, nerve function impairment, leprosy reactions and physical disabilities (Table 1).

The total number of events was 3715 with a mean of 148.6 (maximum: 973 and minimum: 9) within a total population of 85,265. The mean follow-up of outcomes was found in 10 (35.7%) articles with an average of 5.6 years until the events occurred, and the mean follow-up in the following events was identified: leprosy (one article; average 2.3 years), relapse (four articles; average 5.9 years), nerve function impairment (one article; 5.2 years), leprosy reactions (two articles; average 4.9 years), physical disability (four articles; average 8.3 years) and other outcomes (two articles; average 2.8 years). The total number of articles exceeds 10, considering the articles contained more than one outcome or the mean result time contained more than one study group (Table 1). 

In 14 of the studies, the incidence density per 100 person-years was calculated. The methods applied to perform the survival analysis were the Kaplan–Meier method (23; 82.1%), log-rank test (16; 57.1%), Cox’s proportional hazards regression (13; 46.4%) and hazard ratio (14; 50.0%) (Table 1).

#### 3.2.1. Leprosy

The leprosy outcome was found in six articles (21.4%), of which the country locations were: Brazil (3; 50.0%), Indonesia (2; 16.7%) and India (1; 33.3%). The study population consisted of leprosy contacts and the contact population was specified in five articles, two (16.7%) in household and three (50.0%) in household and neighbor (Table 1).

The mean follow-up was found in one article (mean 2.3 years). The incidence density per 100 person-years was calculated in three studies (50.0%). The methods applied to perform the survival analysis were the Kaplan–Meier method (4; 66.7%), log-rank test (4; 66.7%), Cox’s proportional hazards regression (3; 50.0%) and hazard ratio (5; 83.3%) (Table 1).

In household contacts, multibacillary patients have a higher incidence density of 1313/1000 person-years. The risk estimate (hazard ratio) of the disease occurring in the family contacts of multibacillary patients was 4.60 times higher (95% CI 1.65–12.9) than in the non-contact group (Table 2).

#### 3.2.2. Relapse

The relapse outcome was identified in eight articles (28.6%), which were located in the following countries: India (3; 37.5%), Brazil (2; 25.0%), Colombia (1; 12.5%), the Philippines (1; 12.5%) and the Republic of Zaire (now the Republic of Congo) (1; 12.5%). The study population consisted of patients with leprosy, being more frequent in multibacillary patients with four studies (50.0%) (Table 1). 

The mean follow-up was found in four articles (mean 5.9 years). The incidence density per 100 person-years was calculated in six studies (75.0%). The methods applied to perform the survival analysis were the Kaplan–Meier method (8; 100.0%), log-rank test (5; 62.5%), and the regression model was used in three studies: Cox’s proportional hazards regression (1; 12.5%), Andersen–Gill method (1, 12.5%) and negative binomial regression (1; 12.5%) and the measure of association was hazard ratio (2; 25.0%) (Table 1).

In paucibacillary patients, when there was an intervention with more drugs in the treatment, there was a decrease in the incidence density (C4: 1.9/100 person-years; C-ROM: 0.90/100 person-years). In multibacillary patients, those who received 24 doses of the regimen treated to the point of smear negative had an incidence density of 1.11/100 person-years. When comparing paucibacillary and multibacillary patients, the latter group had the highest incidence density, with 0.595/100 person-years. The FDT group multibacillary (24 doses up to the point of smear negative) had a higher probability of no relapse than the NRT (24 doses) multibacillary up to eight years, and the probability of relapse was higher in multi-bacillary patients up to 15 years of age with 12 treatment doses (90.19%) and 24 treatment doses (63.6%) (Table 3).

#### 3.2.3. Clinical Manifestations before, during and after Treatment

Outcomes encompassed in clinical manifestations before, during and after treatment that stood out were located in 14 articles (50.0%), distributed in leprosy reactions (5), nerve function impairment (7) and physical disabilities (5). The number exceeds 14, since the articles contained more than one outcome. The research locations were in Brazil (6; 42.9%), Bangladesh (4; 28.6%), India (2; 14.3%), Bangladesh and Nepal (1; 7.1%) and the Republic of Zaire (now the Republic of Congo) (1; 7.1%). The study population consisted of patients with leprosy, with multibacillary patients prevailing in six studies (42.9%) (Table 1).

The mean follow-up in clinical manifestations outcomes before, during and after leprosy treatment was found in five articles (mean 7.2 years). The incidence density per 100 person-years was calculated in six studies (42.9%). The methods applied to perform the survival analysis were the Kaplan–Meier method (11; 78.6%), log-rank test (9; 64.3%), Cox’s proportional hazards regression (7; 50.0%) and hazard ratio (7; 50.0%) (Table 1).

In leprosy reactions, a shorter mean follow-up was found in paucibacillary patients (six months) than in multibacillary patients (eight months). The probability up to six months of treatment of not occurring leprosy reactions in multibacillary patients with U-MDT (six doses) was higher at 64.14% than in relation to R-MDT (12 doses) at 62.23%. The risk estimate (hazard ratio) of leprosy reactions in the paucibacillary group was 1.244 times higher than in the multibacillary group (Table 4).

The highest incidence density of nerve function impairment was in the multibacillary group, with 24.4/100 person-years. The probability of nerve function impairment occurring up to two years was higher in the multibacillary group (37.0%) than in the paucibacillary group (2.6%). The risk estimate (hazard ratio) of nerve function impairment occurring in the multibacillary group was 8.8 to 7.5 times greater than in the paucibacillary group (Table 5).

The incidence density of physical disabilities in multibacillary patients ranged from 2.74 to 6.5/100 person-years. The multibacillary R-MDT group (12 doses) (33.8%) had a higher probability of physical disabilities than the U-MDT group (six doses) (30.06%) up to five years of treatment. The risk estimate (hazard ratio) of physical disabilities occurring in multibacillary after treatment was 2.80 times higher in those with grade 2 disabilities compared to those with grade 0 (Table 6).

### 3.3. Quality Assessment Criteria

To assess the quality of the studies, the Standard Quality Assessment Criteria for Evaluating Primary Research Papers from a Variety of Fields were used [15]. The quality percentages of studies ranged from 41 to 86%, with a mean of 63.2%. Five (17.9%) studies scored less than 55% on the summary score [8,16,19,26,37]. The studies that varied between 56% and 74% were 20 (71.4%) articles considered to have more complete data compared to the previous score. Whilst three (10.7%) articles had a score above 75%, having better quality information: Penna et al. (2017) [35], Gomes et al. (2019) [36] and Smith et al. (2004) [22]. Five selected articles had scores below 55%, however, these remained because they were inclusive and comprehensive studies. Table 7 shows the breakdown of quality scores for each study.

## 4. Discussion

This systematic review provides an opportune compilation of information on mean time estimates of outcomes of interest for leprosy that will be useful for national programs over the next decade. Considering that the topics discussed in this study seek to aid the strategies to achieve the proposed goal of ending neglected tropical disease epidemics by 2030 according to the third Sustainable Development Goal [41] and the main goal of the Global Leprosy Strategy 2021–2030, which is defined as the interruption of transmission [42].

In this review, 28 articles were analyzed that were published between the years 1994 and the month of January 2022. The study designs were cohorts and clinical trials. The countries with the highest number of studies were Brazil, India, Indonesia, Bangladesh and the Philippines, with the first three countries representing the highest incidence of leprosy in the world [6]. The main outcomes identified in the studies were leprosy, relapse, nerve function impairment, leprosy reactions and physical disabilities. The mean times and outcomes are key elements of discussion for the prevention and control of leprosy [4,39,40,43].

### 4.1. Leprosy

The six studies included in this review with leprosy outcomes were mostly from Brazil, with the study population being leprosy contacts [8] and household contacts [36,37]. This was followed by Indonesia [23,24] and India [25], with the study populations being household and neighbor contacts. According to Quilter et al. (2020) [44], individuals with the most contact with the sick would be the most susceptible to infection. While Romanholo et al. (2018) [45] emphasize that household contacts compose the group most likely to get sick. Corroborating with this systematic review, which identified the highest risk of becoming ill in household contacts [24,25] and specifically for contacts of multibacillary patients [24]. According to Teixeira et al. (2020) [46], contacts who lived with multibacillary patients, aged over 50 years and with schooling up to high school were more likely to develop the disease.

However, non-household spaces such as work and school provide intense inter-human contact [47]. Moreover, other forms of contact besides households can be important in the transmission of the disease [43,47]. A multi-centric study carried out in Brazil, India, Indonesia, Myanmar and Nepal, identified a percentage above 50% of patients with leprosy among social and neighborhood contacts [48]. In China, 69% of leprosy cases were non-household contacts [43]. In this review, the highest incidence density was in household and neighbor contacts with 0.298/100 person-years [24] and the lowest was in the group of non-familial contacts with 0.046/100 person-years [25].

The statistical methods used in the six studies were Kaplan–Meier, identified in four articles which also used the log-rank test [23,25,36,37]. In three studies, Cox’s proportional hazards regression was used [8,23,24], while in five other studies, the hazard ratio was applied [8,23,24,36,37]. The mean time until the development of leprosy was found in only one article, being 1.91 years for those who did not have the Bacille Calmette–Guerin (BCG) vaccine scar, 1.97 years with only one BCG vaccine scar and 3.00 years with two BCG vaccine scars [36]. In the research by Niitsuma et al. (2021) [49], BCG immunization was found to have a protective effect against illness (RR = 0.52; 95% CI 0.34–0.78).

### 4.2. Relapse

The studies included in this review with an outcome of relapse for leprosy are based on the concept of the World Health Organization (WHO): patients treated regularly using the standardized therapeutic regimen according to operational classification, discharged due to cure, and after a period of time, present once again with clinical signs and symptoms of the infectious disease [50].

In this review, three articles were from India with a study population featuring both multibacillary [19,39] and paucibacillary leprosy [30,39]. Two studies were from Brazil [35,40]. The remaining studies from Colombia [31], the Philippines [20] and the Republic of Zaire (now the Republic of Congo) also researched the same population [16].

In surveys carried out in specialized centers for leprosy, the results showed that the percentage of relapse cases in Northern India was 61% [51]; in Northern Italy in refugees/migrants, it was 35% [52] and in Midwest Brazil, it was 10 % [53] Studies carried out in two Brazilian states revealed that the relapse of the disease is more frequent in multibacillary patients [54,55]. Already under study in Colombia, the virchowian clinical form is specified, which is classified as multibacillary, with a four-times greater probability of the disease occurring compared to other clinical forms [31].

Other studies reaffirm that the greatest risk of developing relapse was in patients with multibacillary leprosy when compared to patients with paucibacillary leprosy, which is justifiable due to the high bacillary load [40,56], ratifying what was found in this systematic review [39,40]. However, in this review, the incidence density of relapse in multibacillary patients ranged from 0.28/100 person-years [20] to 6.74/100 person-years [31].

According to Rajkumar et al. (2021) [39], low relapse rates are indicative of treatment effectiveness. However, factors such as adequate and opportune treatment regimens for paucibacillary and multibacillary patients as well as drug resistance should be considered [39]. In this review, it was investigated when treatment was combined with other medications [16,30] or when a longer use of treatment with 24 doses was adopted [40], or in addition to prolonged treatment (24 doses) associated until the point of smear negativity [19], there is a lower risk of relapse.

The statistical methods used in all eight studies were Kaplan–Meier [16,19,20,30,31,35,39,40]. Of these, five articles used the log-rank test [16,30,31,35,40], three used regression models: Cox’s proportional hazards regression [31], negative binomial regression [35] and the Andersen–Gill method [39], and two studies applied the hazard ratio [31,39]. The mean time to relapse was identified in four articles, the longest at 11.6 years [40] and the shortest at 1.6 years in paucibacillary patients undergoing ROM treatment [30]. There are differences among authors regarding the time to relapse, with some considering relapse at any time after the patient is discharged from treatment [31,57,58], and others emphasizing a minimum interval of three years [59], five years [40,60,61] and up to 16 years [55,62]. It is worth noting that WHO [50] considers the time until relapse to be three years for paucibacillary patients and five years for multibacillary patients.

Gonçalves et al. (2019) [63] state that relapses greater than 15 years are commonly experienced by those who are continuously exposed to the bacillus. Chagas et al. (2021) [55], ratify this result, as 40% of relapses in their patients were due to household contact. Lobo et al. (1992) [64] advise about their finding of 24% of relapses detected in the first 18 months, as they may be late reversal reactions. In the work of Nascimento et al. (2022) [40], the importance of identified cases of relapses to immediately starting multidrug therapy is highlighted in order to avoid physical disabilities and the proliferation of the disease.

### 4.3. Clinical Manifestations before, during and after Treatment

In this review, in order to carry out the discussion, the outcomes with clinical manifestations that can occur before, during and after multidrug therapy were grouped [7,65]. These manifestations are derived from the bacillary load and the immune response to the etiological agent, Mycobacterium leprae, which is likely to affect between 30 and 50% of leprosy patients [66,67]. The grouping was carried out according to the outcomes: leprosy reactions and nerve function impairment that are interconnected and influence the final event, physical disabilities [7,68].

Among the findings, 14 articles presented one or more of these outcomes: five of leprosy reactions, seven nerve function impairments and five of physical disabilities. Most were from Brazil, the study population being patients with paucibacillary [26], multibacillary [33,34,35] leprosy, leprosy reaction during and after multidrug therapy [38] and cured [7]. Four articles in Bangladesh evaluated the general population with leprosy [17,18,21,28], while a study carried out in Bangladesh and Nepal was performed on multibacillary patients [22]. This last study’s population was the same investigated in India [29,32]. In the Republic of Zaire (now the Republic of Congo), a study was carried out with paucibacillary patients [16].

In studies carried out in specialized leprosy centers in Brazil, it was stated that leprosy reactions occur in approximately 10 to 50% of multibacillary patients [69,70]. On the other hand, this systematic review identified greater vulnerability to leprosy reactions in paucibacillary patients [38]. In a multi-centric research carried out at reference centers in India, Nepal, Bangladesh and Indonesia, the nerve function impairment occurred mainly in multibacillary patients with reactions [71]. In this review, it was found that multibacillary patients are at greater risk of having nerve function impairment [17,18,21,28] and physical disabilities are more favorable to multibacillary patients who have developed disability grade 2 [34]. Worldwide, the number of new cases of leprosy with grade 2 disability represents 7214; with a proportion of new cases with grade 2 disability of 5.66% [6].

Santos et al. (2020) [7] state that the risk of the progression of physical disabilities is associated with leprosy reactions, especially those reported during multidrug therapy. A Brazilian study identified that leprosy reactions are more frequent during multidrug therapy; however, approximately a third occurred after treatment [38]. It was also identified in other studies that leprosy reactions occur in about 15% to 23% of cases after multidrug therapy [72,73]. As for nerve function impairment, the risk of developing this clinical manifestation can reach up to 65% within two years after the start of treatment [74]. Other researchers observed that patients treated and cured after 10 and 15 years had a 30% and 35% probability of progressing to physical disability, respectively [7,75]. In this review, the highest incidence density was of multibacillary leprosy patients with physical disability outcome post multidrug therapy with 6.5/100 person-years [34].

The statistical methods used in 11 articles were Kaplan–Meier [7,16,18,21,26,28,29,33,34,35,38]. Of these, the log-rank was performed in seven articles [7,16,17,26,33,35,38]. Cox’s proportional hazards regression was used in seven articles [7,17,18,28,29,34,38], multivariable Poisson regression was used in one article [33] and negative binomial regression was used in one article [35]. Regarding the association measures, the hazard ratio was applied most as it was used in seven articles [7,17,21,28,29,34,38], and the odds ratio [32] and relative risk [22,33] were also used.

The mean time until the onset of physical disabilities was shorter in multibacillary patients, averaging 4.28 years [32] and longer in paucibacillary patients, averaging 13.5 years [7]. This occurs because the bacillary load of multibacillary leprosy is higher [3]. However, in a cohort of multibacillary patients, there was a 40% progression of physical disability in patients after 10 years of completion of multidrug therapy [34]. Research in India [76], Nigeria [77] and Brazil [5,78] indicates the development of physical disabilities after the end of treatment.

Early detection and treatment are priority strategies for reducing physical disabilities [5,39,79], which can be irreversible and cause emotional, social and economic damage to patients [5,79]. Preventing these disabilities is one of the goals of the Global Leprosy Strategy 2021–2030 [42]. In this way, the implementation of surveillance of physical disabilities with the systematic follow-up of patients during and after completion of treatment is suggested [38,80].

In order to support the development of adequate surveillance in leprosy patients, monitoring and evaluating probable cases of relapses and physical disabilities is necessary, especially in multibacillary patients, since they have the highest load of Mycobacterium leprae. Another priority would be to investigate household and non-household contacts encouraging BCG vaccination.

On the other hand, for the evaluation and monitoring of national leprosy programs, it is recommended to include the mean time until the final outcomes in the epidemiological indicators. In addition, insert the incidence density, the use of Kaplan–Meier methods, log-rank test, Cox’s proportional hazards regression and the hazard ratio [9,10,81].

In this way, these indicators will enable the evaluation of the leprosy control program in the following aspects: (I) leprosy in contacts: will evaluate the time of services to identify new cases between household and non-household contacts; (II) relapse: it will evaluate the duration of the therapeutic regimen and the effectiveness of the treatment; (III) clinical manifestations before, during and after treatment: it will evaluate the time until the appearance of physical deformities and consequently will assess the activities of opportune and/or early detection of cases in the health services.

These indicators described above aim to contribute to the Global Leprosy Strategy 2021–2030 by reducing the number of new cases and new cases detected with grade-2 disabilities [42]. The (I) leprosy in contacts can be an aggregate indicator in the second strategic pillar “scale up leprosy prevention alongside integrated active case detection”, and the (III) clinical manifestations before, during and after treatment can be aggregated in the third strategic pillar “manage leprosy and its complications and prevent new disability”. The (II) relapse is not addressed in the overall strategy but is a relevant indicator to evaluate drug therapy. The use of survival analysis resources has been shown to be a valuable tool for monitoring and controlling leprosy.

Regarding the quality of the analyzed articles, most of them (82.10%) are of high and medium quality. Thus, we can assume that the data extracted from them reflects the reality of the study areas. Other systematic reviews about leprosy have also included high-quality studies in their work [49,82,83]. Despite 17.9% of the studies included in our systematic review being evaluated as low-quality, it does not affect the overall standard of this research.

### 4.4. Limitations

Due to the heterogeneity of the study methods and the variation in mean time and incidence density, it was not possible to perform a meta-analysis of the results. As a limitation, articles in English and Portuguese were included, as the disease is found mostly in countries that mainly use these languages.

## 5. Conclusions

This is the first review to systematically investigate the mean time taken place until the outcomes: (I) leprosy, (II) relapse and (III) clinical manifestations before, during and after treatment. The mean time for the development of leprosy in those who were exposed was longer for those who had more than one dose of BCG. In the case of relapse, there were discrepancies between the authors regarding the mean time to this outcome; however, short periods must be given attention, as it can be mistaken with late reverse reactions. In clinical manifestations, the mean time was longer for paucibacillary patients compared to multibacillary patients. These last patients were highlighted in this review, and they need to be prioritized, given that the Global Leprosy Strategy 2021–2030 aims to interrupt the transmission of the disease. Therefore, the use of survival analysis will make it possible to evaluate national programs. In this way, it will assist in the decision-making process to face public health problems, affecting the quality of health services provided to patients affected by leprosy.

## Figures and Tables

**Table 1:** Reporting survival analyses of the included studies.

Study/Year of Publication	Study Design	Country	Follow Up Duration (Years)	Study Population (Total)	Main Outcome (Event)	Event/Total	Mean Follow-Up (Years)	Incidence Density/100 Person-Years	Survival Curve	Survival Curve Comparison Method	Regression Model	Measures of Association/Effect
Pattyn et al. (1994) ^a^ [16]	Clinical trial	The Republic of Zaire (now the Republic of Congo)	4	Leprosy patients/paucibacillary	Cure/Relapse/Grade 2 disability	Cure: C2: 214/317; C4: 206/305, Relapse: C2: 9/317; C4: 6/305, Grade 2 disability: C2: 175/317; C4: 157/305	NA	Relapse: C2: 3.3; C4: 1.9	Kaplan–Meier method	log-rank test	NA	Relative Risk
Croft, Nicholls, Richardus et al. (2000) [17]	Cohort	Bangladesh	2	Leprosy patients	Nerve Function Impairment	166/2510	NA	3.7	NA	log-rank test	Cox’s proportional hazards regression	Hazard Ratio
Croft, Nicholls, Steyerberg et al. (2000) [18]	Cohort/prospective	Bangladesh	2	Leprosy patients	Nerve Function Impairment	166/2510	NA	NA	Kaplan–Meier method	NA	Cox’s proportional hazards regression	Hazard Ratio
Girdhar et al. (2000) ^b^ [19]	NA	India	FDT: 3, TNS: 4	Leprosy patients/multibacillary	Relapse	FDT: 20/260, TSN: 12/301	NA	FDT: 2.04, TSN: 1.11	Kaplan–Meier method	NA	NA	NA
Cellona et al. (2003) [20]	Cohort/prospective	The Philippines	16	Leprosy patients/multibacillary/cured/after the use of multidrug therapy	Relapse	15/500	10.8	0.28	Kaplan–Meier method	NA	NA	NA
Richardus et al. (2004) [21]	Cohort/prospective	Bangladesh	5	Leprosy patients	Nerve Function Impairment	Paucibacillary: 54/2153, Multibacillary: 121/357	NA	Paucibacillary: 0.85, Multibacillary: 16.1	NA	log-rank test	NA	NA
Smith et al. (2004) ^c^ [22]	Clinical trial	Bangladesh and Nepal	3	Leprosy patients/multibacillary	Nerve Function Impairment	153/636	NA	NA	Kaplan–Meier method	NA	NA	Relative Risk
Bakker et al. (2005) ^d^ [23]	Clinical trial	Indonesia	4	Leprosy contacts/household/neighbor	Leprosy	Control group: 11/1252, Contact group: 15/1633, Blanket group: 3/1080	NA	NA	Kaplan–Meier method	log-rank test	Cox’s proportional hazards regression	Hazard Ratio
Bakker et al. (2006) ^e^ [24]	Cohort	Indonesia	4	Leprosy contacts/household/neighbor	Leprosy	44/4903	NA	0.298	NA	NA	Cox’s proportional hazards regression	Hazard Ratio
Kumar et al. (2007) ^f^ [25]	NA	India	4	Leprosy contacts/household/neighbor	Leprosy	77/42,113, non-familial contacts: 56/41.119, familial contacts: 21/994	NA	0.062, non-familial contacts: 0.046, familial contacts: 0.676	Kaplan–Meier method	log-rank test	NA	NA
Gomes et al. (2008) ^g^ [26]	Cohort/prospective	Brazil	3	Leprosy patients/paucibacillary	Leprosy reactions/Neuritis/Onset and increase of new wounds/Change of operational classification.	46/259	NA	0.069/100 person-months	Kaplan–Meier method	log-rank test	NA	NA
Gonçalves et al. (2008) [27]	Cohort/Retrospective	Brazil	11	Leprosy patients	Neuritis	281/529	NA	NA	Kaplan–Meier method	log-rank test	Cox’s proportional hazards regression	Hazard Ratio
Schuring et al. (2008) ^h^ [28]	Cohort/prospective	Bangladesh	4	Leprosy patients	Nerve Function Impairment	115/864	NA	NA	Kaplan–Meier method	NA	Cox’s proportional hazards regression	Hazard Ratio
Smith et al. (2009) [29]	Cohort	India	2	Leprosy patients/multibacillary	Nerve Function Impairment/Leprosy reactions	74/188	NA	NA	Kaplan–Meier method	NA	Cox’s proportional hazards regression	Hazard Ratio
Girdhar et al. (2011) ^i^ [30]	Clinical trial	India	5	Leprosy patients/paucibacillary	Relapse	9/300, ROM: 05/151, C-ROM: 04/149	ROM: 1.6 C-ROM: 1.7	ROM: 1.05, C-ROM: 0.90	Kaplan–Meier method	log-rank test	NA	NA
Guerrero-Guerrero et al. (2012) [31]	Cohort/Retrospective	Colombia	11	Leprosy patients/multibacillary	Relapse	33/243	NA	6.74	Kaplan–Meier method	log-rank test	Cox’s proportional hazards regression	Hazard Ratio
Kumar et al. (2012) [32]	Cohort/prospective	India	6	Leprosy patients/multibacillary	Physical disability pre-multidrug therapy e post-multidrug therapy	24/205	4.28	2.74	NA	log-rank test	NA	Odds Ratio
Penna et al. (2012) ^j^ [33]	Clinical trial	Brazil	6	Leprosy patients/multibacillary	Nerve Function Impairment/Leprosy reactions	U-MTD: 120/306, R-MDT: 90/272	First leprosy reactions: 5.2	NA	Kaplan–Meier method	log-rank test	Multivariable Poisson regression	Relative Risk
Sales et al. (2013) [34]	Cohort	Brazil	14	Leprosy patients/multibacillary	Physical disability post-multidrug therapy	103/368	4.3	6.5	Kaplan–Meier method	NA	Cox’s proportional hazards regression	Hazard Ratio
Penna et al. (2017) ^j^ [35]	Clinical trial	Brazil	8	Leprosy patients/multibacillary	Leprosy reactions/Physical disability/Bacilloscopic index (≥4 and <4)/Relapse	U-MTD: NA/323, R-MDT: NA/290	U-MTD: 4.86, R-MDT: 4.77	NA	Kaplan–Meier method	log-rank test	Negative binomial regression	NA
Gomes et al. (2019) [36]	Cohort/Retrospective	Brazil	16	Leprosy contacts/household	Leprosy	92/5061	without BCG vaccine scar: 1.91, one BCG vaccine scar: 1.97, two BCG vaccine scars: 3.00	NA	Kaplan–Meier method	log-rank, Breslow, and Tarone–Ware tests	NA	Relative Risk and Hazard Ratio
Manta et al. (2019) [37]	Cohort	Brazil	7	Leprosy contacts/household	Leprosy	69/2437	NA	NA	Kaplan–Meier method	log-rank test	NA	Hazard Ratio
Santos et al. (2020) ^k^ [7]	Cohort/Retrospective	Brazil	17	Leprosy patients/cured	Physical disability post-multidrug therapy	188/385	Paucibacillary: 13.5. multibacillary 12.6, Leprosy reactions: 10.8, reports of complaints during treatment 11.6	NA	Kaplan–Meier method	log-rank test	Cox’s proportional hazards regression	Hazard Ratio
Coriolano et al. (2021) [38]	Cohort	Brazil	9	Leprosy patients/leprosy reactions during and after the use of multidrug therapy	First leprosy reactions during multidrug therapy and post-multidrug therapy	NA/1621	NA	NA	Kaplan–Meier method	log-rank test	Cox’s proportional hazards regression	Hazard Ratio
Hacker et al. (2021) ^l^ [8]	Cohort	Brazil	33	Leprosy contacts	Leprosy	192/9024	NA	0.141	NA	NA	Cox’s proportional hazards regression	Hazard Ratio
Pepito et al. (2021) ^m^ [11]	Cohort/Retrospective	The Philippines	7	Leprosy patients/multibacillary	Treatment completion/Treatment default	Treatment completion: 590/1034, Treatment default: 383/1034	Treatment completion: 1.1, Treatment default: 0.3	NA	Kaplan–Meier method	log-rank test	Cox’s proportional hazards regression	NA
Rajkumar et al. (2021) ^n^ [39]	Cohort	India	10	Leprosy patients/cured	Relapse	69/2177	NA	0.542	Kaplan–Meier method	Mid-p exact test	Andersen–Gill method	Hazard Ratio
Nascimento et al. (2022) ^o^ [40]	Retrospective	Brazil	6	Leprosy patients/cured	Relapse	126/1059	11.6	NA	Kaplan–Meier method	log-rank, Breslow and Tarone–Ware tests.	NA	NA

NA—Not available. ^a^ The two single-dose treatment regimens (adult doses) were: C2: 40 mg/kg rifampicin (RMP) and 1200 mg clofazimine (CLO); C4: 40 mg/kg RMP, 100 mg CLO, 100 mg dapsone (DDS) and 500 mg ethionamide (ETH). ^b^ FDT: given therapy for a fixed duration (24 doses of WHO MB regimen); TNS: treated with the same regimen but up to the point of smear negativity. Duration of follow-up years: FDT: mean 2.7 ± 1.4 years; TNS: average 3.82 ± 2.79 years. ^c^ Clinical trial using prednisolone for the prevention of nerve function impairment. ^d^ Clinical trial using rifampicin as a prophylactic measure in leprosy control. Control group: did not use rifampicin, contact group: rifampicin was administered in one study location, blanket group: prophylaxis was applied in three study locations. Duration of follow-up years: 33.5 months. ^e^ Incidence density 2.98 per 1000 persons-year. ^f^ Incidence density: 6.2/10,000 persons-year; non-familial contacts—4.6/10,000 persons-year; familial contacts 67.6/10,000 persons-year. ^g^ Incidence density: 6.9 per 1000 persons-month. ^h^ Follow-up duration: 46 months approximately 3.8 years. ^i^ Evaluating the effects of clarithromycin on rifampicin, ofloxacin, and minocycline in the treatment of paucibacillary leprosy. ROM: Rifampicin, Ofloxacin and Minocycline; C-ROM: Clarithromycin, Rifampicin, Ofloxacin and Minocycline. Mean follow-up in ROM: 19.3 months, C-ROM: 19.8 months. ^j^ Evaluation of multidrug therapy (MDT) with three drugs—rifampicin, dapsone and clofazimine, in two patient groups MB, uniform treatment (U-MTD) for 6 months and R-MDT for 12 months according to the WHO recommendation. ^k^ Mean follow-up for physical disability post multidrug-therapy in paucibacillary patients: 162 months, multibacillary: 151 months, leprosy reactions 130 months, reports of complaints during treatment: 139 months. ^l^ Incidence density: 1.4/1000 persons-year. ^m^ Mean follow-up: Completion of treatment: 13.4 months, Dropout from treatment: 3.6 months. ^n^ Incidence density: 5.42 per 1000 persons-year ^o^ Mean follow-up: 139.156 months.

**Table 2:** Reporting survival analyses of the included studies by outcome leprosy household contact.

Study/Year of Publication	Outcome: Leprosy Household Contact							
	Paucibacillary				Multibacillary			
	Mean Follow-Up (Years)	Incidence Density/100 Person-Years (95% CI)	Curve Kaplan Meier (Years)	Hazard Ratio (95% CI)	Mean Follow-Up (Years)	Incidence Density/100 Person-Years (95% CI)	Curve Kaplan–Meier (Years)	Hazard Ratio (95% CI)
Bakker et al. (2005) [23]	NA	NA	NA	NA	NA	NA	NA	NA
Bakker et al. (2006) ^a^ [24]	233	0.215 (0.030–1.52)	NA	No contact-1.0; 0.97 (0.13–7.32)	217	1.15 (0.480–2.77)	NA	No contact-1.0; 4.60 (1.65–12.9)
Kumar et al. (2007) ^b^ [25]	NA	0.410 (NA)	NA	NA	NA	1.313 (NA)	NA	NA
Gomes et al. (2019) [36]	NA	NA	NA	NA	NA	NA	NA	NA
Manta et al. (2019) [37]	NA	NA	NA	NA	NA	NA	NA	NA
Hacker et al. (2021) [8]	NA	NA	NA	NA	NA	NA	NA	NA

NA—Not available. ^a^ Paucibacillary: Follow up time 5592.8 months; incidence rate 2.15/1000 (95% CI: 0.30–15.2) person-years. Multibacillary: Follow up time 5201.0 months; incidence rate 11.5/1000 (95% CI: 4.80–27.7) person-years. ^b^ Paucibacillary: Incidence rate 41.0/10,000 person-years. Multibacillary: incidence rate 131.3/10,000 person-years.

**Table 3:** Reporting survival analyses of the included studies by outcome relapse.

Study/Year of Publication	Outcome: Relapse							
	Paucibacillary				Multibacillary			
	Mean Follow-Up (Years)	Incidence Density/100 Person-Years (95% CI)	Curve Kaplan–Meier (Years)	Hazard Ratio (95% CI)	Mean Follow-Up (Years)	Incidence Density/100 Person-Years (95% CI)	Curve Kaplan–Meier (Years)	Hazard Ratio (95% CI)
Pattyn et al. (1994) ^a^ [16]	NA	C2: 3.3 (1.1–5.4); C4: 1.9 (0.7–4.0)	NA	NA	NA	NA	NA	NA
Girdhar et al. (2000) ^b^ [19]	NA	NA	NA	NA	NA	FDT: 2.04 (NA); TSN: 1.11 (NA)	FDT < 90.0% (8); TSN < 100.0% (8) relapse-free	NA
Cellona et al. (2003) [20]	NA	NA	NA	NA	NA	0.28	4.0% (12)	NA
Girdhar et al. (2011) ^c^ [30]	ROM: 1.6 C-ROM: 1.7	ROM: 1.05 (NA), C-ROM: 0.90 (NA)	NA	NA	NA	NA	NA	NA
Guerrero-Guerrero et al. (2012) [31]	NA	NA	NA	NA	NA	6.70	<75.0% (10)	NA
Penna et al. (2017) ^d^ [35]	NA	NA	NA	NA	NA	U-MDT: 0.446 (NA); R-MDT: 0.044 (NA)	NA	NA
Rajkumar et al. (2021) ^e^ [39]	NA	0.506 (NA)	NA	NA		0.595 (NA)	NA	NA
Nascimento et al. (2022) ^f^ [40]	10	NA	MDT-PB 6 dose: 64.28% (10); 85.71% (15)	NA	14	NA	MDT-MB 12 dose: 70.58% (10); 90.19% (15) MDT-MB 24 dose: 38.6% (10); 63.6% (15)	NA

NA—Not available. ^a^ The two single-dose treatment regimens (adult doses) were: C2: 40 mg/kg rifampicin (RMP) and 1200 mg clofazimine (CLO); C4: 40 mg/kg RMP, 100 mg CLO, 100 mg dapsone (DDS) and 500 mg ethionamide (ETH). ^b^ FDT: given therapy for a fixed duration (24 doses of WHO MB regimen); TNS: treated with the same regimen but up to the point of smear negativity. ^c^ Evaluating the effect of clarithromycin on rifampicin, ofloxacin and minocycline in the treatment of paucibacillary leprosy. ROM: Rifampicin, Ofloxacin and Minocycline; C-ROM: Clarithromycin, Rifampicin, Ofloxacin and Minocycline. ^d^ Evaluation of multidrug therapy (R-MDT) with three drugs—rifampicin, dapsone and clofazimine, in two patient groups MB, uniform treatment (U-MTD) for 6 months and R-MDT for 12 months according to the WHO recommendation. Rate of relapse for the U-MDT group was 4.46 per 1000 people-year and for R-MDT it was 0.44 per 1000 people-year. 1825 days. ^e^ Relapse rate paucibacillary: 5.06/1000 person-years Relapse rate multibacillary: 5.95/1000. ^f^ Paucibacillary: Length of time mean until relapse 118.286 (in months); multibacillary: Length of time mean until relapse of multidrug therapy (MDT) 12 dose: 117.176 (in months) and multidrug therapy 24 dose: 171.273 (in months).

**Table 4:** Reporting survival analyses of the included studies by outcome clinical manifestations before, during and after treatment—leprosy reactions.

Study/Year of Publication	Outcome: Clinical Manifestations before, during and after Treatment—Leprosy Reactions							
	Paucibacillary				Multibacillary			
	Mean Follow-Up (Years)	Incidence Density/100 Person-Years (95% CI)	Curve Kaplan–Meier (Years)	Hazard Ratio (95% CI)	Mean Follow-Up (Years)	Incidence Density/100 Person-Years (95% CI)	Curve Kaplan–Meier (Years)	Hazard Ratio (95% CI)
Gomes et al. (2008) [26]	NA	NA	NA	NA	NA	NA	NA	NA
Smith et al. (2009) [29]	NA	NA	NA	NA	NA	NA	NA	NA
Penna et al. (2012) ^a^ [33]	NA	NA	NA	NA	5.2	NA	NA	NA
Penna et al. (2017) ^a^ [35]	NA	NA	NA	NA	NA	NA	U-MDT: 64.14% (6 months) R-MDT: 62.23% (6 months) reaction-free	NA
Coriolano et al. (2021) [38]	6 months	NA	NA	1.244 (1.108–1.397)	8 months	NA	NA	1.0

NA—Not available. ^a^ Evaluation of multidrug therapy (R-MDT) with three drugs—rifampicin, dapsone and clofazimine, in two patient groups MB, uniform treatment (U-MTD) for 6 months and R-MDT for 12 months according to the WHO recommendation.

**Table 5:** Reporting survival analyses of the included studies by outcome clinical manifestations before, during and after treatment—nerve function impairment.

Study/Year of Publication	Outcome: Clinical Manifestations before, during and after Treatment—Nerve Function Impairment							
	Paucibacillary				Multibacillary			
	Mean Follow-Up (Years)	Incidence Density/100 Person-Years (95% CI)	Curve Kaplan–Meier (Years)	Hazard Ratio (95% CI)	Mean Follow-Up (Years)	Incidence Density/100 Person-Years (95% CI)	Curve Kaplan–Meier (Years)	Hazard Ratio (95% CI)
Croft, Nicholls, Richardus et al. (2000) [17]	NA	1.3	NA	1.0	NA	24.4	NA	8.8 (6.2–12.5)
Croft, Nicholls, Steyerberg et al. (2000) [18]	NA	NA	2.6% (2)	1.0	NA	NA	37.0% (2)	7.5 (5.3–11.0)
Richardus et al. (2004) [21]	NA	0.85	NA	NA	NA	16.1	NA	NA
Smith et al. (2004) [22]	NA	NA	NA	NA	NA	NA	NA	Relative Risk: 2.0 (0.8–4.5)
Schuring et al. (2008) [28]	NA	NA	NA	1.0	NA	NA	NA	8.0 (5.0–13.0)
Smith et al. (2009) [29]	NA	NA	NA	NA	NA	NA		NA
Penna et al. (2012) [33]	NA	NA	NA	NA	NA	NA	NA	NA

NA—Not available.

**Table 6:** Reporting survival analyses of the included studies by outcome clinical manifestations before, during and after treatment—physical disabilities.

Study/Year of Publication	Outcome: Clinical Manifestations before, during and after Treatment—Physical Disabilities							
	Paucibacillary				Multibacillary			
	Mean Follow-Up (Years)	Incidence Density/100 Person-Years (95% CI)	Curve Kaplan–Meier (Years)	Hazard Ratio (95% CI)	Mean Follow-Up (Years)	Incidence Density/100 Person-Years (95% CI)	Curve Kaplan–Meier (Years)	Hazard Ratio (95% CI)
Pattyn et al. (1994) ^a^ [16]	NA	NA	NA	Relative Risk: C2: 1.0; C4: 1.6 (0.9–3.0) physical disability-free	NA	NA	NA	NA
Kumar et al. (2012) [32]	NA	NA	NA	NA	NA	2.74	NA	NA
Sales et al. (2013) [34]	NA	NA	NA	NA	4.3	6.5	<60.0% (10)	Grade 0: 1.0; Grade 1: 2.03 (1.32–3.14); Grade 2: 2.80 (1.65–4.74)
Penna et al. (2017) ^b^ [35]	NA	NA	NA	NA	NA	NA	U-MDT: 33.8% (5) R-MDT: 30.06% (5)	NA
Santos et al. (2020) ^c^ [7]	NA	NA	< 80.0% (17)	1.0	12.6	NA	<80.0% (17)	0.82 (0.60–1.11)

NA—Not available. ^a^ The two single-dose treatment regimens (adult doses) were: C2: 40 mg/kg rifampicin (RMP) and 1200 mg clofazimine (CLO); C4: 40 mg/kg RMP, 100 mg CLO, 100 mg dapsone (DDS) and 500 mg ethionamide (ETH). ^b^ Evaluation of multidrug therapy (R-MDT) with three drugs—rifampicin, dapsone and clofazimine, in two patient groups MB, uniform treatment (U-MTD) for 6 months and R-MDT for 12 months according to the WHO recommendation. ^c^ Mean follow-up: 162 months for paucibacillary and 151 months for multibacillary.

**Table 7:** Quality evaluation of the included studies.

Studies	Criteria																
	Question/Objective Sufficiently Described?	Study Design Evident and Appropriate?	Method of Subject/Comparison Group Selection or Source of Information/Input Variables Described and Appropriate?	Subject (and Comparison Group, if Applicable) Characteristics Sufficiently Described?	If Interventional and Random Allocation Was Possible, Was It Described?	If Interventional and Blinding of Investigators Was Possible, Was It Reported?	If Interventional and Blinding of Subjects Was Possible, Was It Reported?	Outcome and (If Applicable) Exposure Measure(s) Well Defined and Robust to Measurement/Misclassification Bias? Means of Assessment Reported?	Sample Size Appropriate?	Analytic Methods Described/Justified and Appropriate?	Some Estimate of Variance Is Reported for the Main Results?	Controlled for Confounding?	Results Reported in Sufficient Detail?	Conclusions Supported by the Results?	Maximum Points	Total Points	Summary Score (%)
Pattyn et al. (1994) [16]	2	2	2	2	1	0	0	0	0	2	1	0	2	1	28	15	53.6
Croft, Nicholls, Steyerberg et al. (2000) [17]	2	2	2	2	N/A	N/A	N/A	2	0	2	1	0	2	1	22	16	72.7
Croft, Nicholls, Richardus et al. (2000) [18]	1	2	2	2	N/A	N/A	N/A	0	0	2	1	0	2	1	22	13	59.1
Girdhar et al. (2000) [19]	2	1	2	2	0	0	0	0	0	2	0	0	2	2	28	13	46.4
Cellona et al. (2003) [20]	2	2	2	2	N/A	N/A	N/A	0	0	2	1	0	2	2	22	15	68.2
Richardus et al. (2004) [21]	2	2	2	2	N/A	N/A	N/A	0	0	2	0	0	2	2	22	14	63.6
Smith et al. (2004) [22]	2	2	2	2	2	2	2	1	2	2	1	0	2	2	28	24	85.7
Bakker et al. (2005) [23]	2	2	1	1	1	1	1	2	0	2	1	0	2	2	28	18	64.3
Bakker et al. (2006) [24]	2	2	1	1	1	1	1	2	0	2	1	0	2	2	28	18	64.3
Kumar et al. (2007) [25]	2	2	2	1	N/A	N/A	N/A	0	2	2	1	0	2	2	22	16	72.7
Gomes et al. (2008) [26]	1	0	1	1	N/A	N/A	N/A	0	1	1	0	0	2	2	22	9	40.9
Gonçalves et al. (2008) [27]	2	2	2	1	N/A	N/A	N/A	0	0	2	1	0	2	2	22	14	63.6
Schuring et al. (2008) [28]	2	1	2	1	N/A	N/A	N/A	0	0	2	1	0	2	2	22	13	59.1
Smith et al. (2009) [29]	2	1	2	1	N/A	N/A	N/A	0	2	2	1	0	2	2	22	15	68.2
Girdhar et al. (2011) [30]	2	2	2	2	1	0	0	0	2	2	0	0	2	2	28	17	60.7
Guerrero-Guerrero et al. (2012) [31]	1	2	2	1	N/A	N/A	N/A	0	0	2	2	0	2	1	22	13	59.1
Kumar et al. (2012) [32]	2	1	2	2	N/A	N/A	N/A	1	0	2	1	0	2	2	22	15	68.2
Penna et al. (2012) [33]	1	2	2	1	1	1	2	0	0	2	1	0	2	1	28	16	57.1
Sales et al. (2013) [34]	2	2	2	1	N/A	N/A	N/A	0	0	2	1	0	2	2	22	14	63.6
Penna et al. (2017) [35]	1	2	2	2	2	2	2	0	2	2	1	0	2	1	28	21	75.0
Gomes et al. (2019) [36]	2	2	2	1	N/A	N/A	N/A	1	2	2	1	0	2	2	22	17	77.3
Manta et al. (2019) [37]	2	2	2	2	N/A	N/A	N/A	0	0	1	0	0	2	1	22	12	54.5
Santos et al. (2020) [7]	2	2	2	1	N/A	N/A	N/A	0	0	2	1	0	2	2	22	14	63.6
Coriolano et al. (2021) [38]	2	2	2	2	N/A	N/A	N/A	1	0	2	1	0	2	1	22	15	68.2
Hacker et al. (2021) [8]	2	1	2	1	N/A	N/A	N/A	0	0	1	1	0	2	2	22	12	54.5
Pepito et al. (2021) [11]	2	2	2	1	N/A	N/A	N/A	0	0	2	1	0	2	2	22	14	63.6
Rajkumar et al. (2021) [39]	2	2	2	1	N/A	N/A	N/A	0	0	2	1	0	2	2	22	14	63.6
Nascimento et al. (2022) [40]	2	1	2	2	N/A	N/A	N/A	0	0	2	1	0	2	1	22	13	59.1

0 if the response is “no”; 1 if the response is “partial”; 2 if the response is “yes”; followed by N/A if not applicable.

## Data Availability

The data presented in this study are available in the present article and its Supplementary Materials reported above.

## Supplementary Materials

The following supporting information can be downloaded at: https://www.mdpi.com/article/10.3390/ijerph191912155/s1, Table S1: PRISMA Checklist.
